# Supplementary material for: A Co-Created Tool to Help Counter Health Misinformation for Spanish-Speaking Communities in the San Francisco Bay Area
Source: Int J Environ Res Public Health. 2024 Mar 2;21(3):294. doi: 10.3390/ijerph21030294 (PMC10970482; doi:10.3390/ijerph21030294)
Supplement: Supplementary file 1 [file ijerph-21-00294-s001.zip › ijerph-2889477-supplementary.pdf]

Supplemental table S1. Main themes, subthemes, and quotes from formative interviews.

Theme	Subtheme	Quote
Impact of Misinformation	Misinformation as a Barrier to Vaccination	"There's a lot of… misinformation. And there is misinformation about any health topic, like this was before the pandemic there is… go to sleep with your phone, … this is how you'll get like, cancer, this is how you'll get like a nosebleed. … there's always been misinformation, but now it's like, through the roof." (KI,06)
	Misinformation's Influence on Vaccine Hesitancy	"We have been bombarded with a lot of misinformation or untruthful information that has frightened us. I had to get COVID twice to get the vaccine. Because I was more scared about the vaccine than COVID. I think that's why a lot of us are reluctant to get the vaccine or we're sensitive to getting untruthful information". (GI,01)
Prevalence and Sources of Misinformation	Prevalence of Misinformation and Its Disproportionate Impact on Spanish Speakers	"In English language, if you put something COVID, fake news, you were over 70% likely to get that tag as false. And if you did the same thing in Spanish, it was like less than 30% likely to be false" (KI,04)
Content of COVID-19 misinformation and myths	Lack of Trust in the FDA Approval Process and Changing Guidelines	"The hard part was trying to tease apart that myth that it wouldn't work well, or what side effects it could, you know, bring about, and I think that was across the board. It wasn't just age-related or race-related, it was everyone." (KI,07)
	Concerns about the Speed of Vaccine Development	"The vaccine is supposedly new; I think that's also where a lot of misinformation comes from. Because we've been put in a lot of fear about the vaccine. It was developed in such a short time they're going to come out with a vaccine. Why haven't they come out for AIDS, for cancer? Why don't they cure those diseases?" (GI,P1)
	Vaccine's Potential Impact on Fertility and Reproductive Health	"So when I take this vaccine, if I were to hypothetically take it, it's gonna be in my body forever. And I just don't know how I feel about that, especially being pregnant, I don't know what that's gonna do to my baby. I've heard like, oh, your baby will be stillborn. And I don't know what if I'm that rare case to where I just die instantly, or the baby dies instantly." (GI, P1)
	Men's Hesitancy Due to Fears of Infertility and Doubts About Their Manhood	"Their big hesitancy, and most of it were men, [was] that they were hearing myths about fertility and that they would become infertile. And I don't even know if it was just infertility. But you know, would they not be able to be manly, that somehow it would affect their manhood." (KI, 08)
